# Traumatic Hemothorax With Rib Fixation in a Professional Jockey Following a Horse Stomping Incident

**DOI:** 10.7759/cureus.75349

**Published:** 2024-12-08

**Authors:** José R Cervantes-Sevilla, Marcela Ramírez-Cervera, Mario J Guzman-Ruvalcaba

**Affiliations:** 1 Faculty of Medicine, Universidad de Guadalajara, Guadalajara, MEX; 2 Faculty of Medicine, Carl von Ossietzky University of Oldenburg, Oldenburg, DEU

**Keywords:** ards, horse stomping, rib fixation, thoracic trauma, traumatic hemothorax

## Abstract

Traumatic hemothorax is a serious condition requiring immediate intervention. We present a case of a 48-year-old male professional jockey who suffered traumatic hemothorax, bilateral pulmonary contusions, and multiple rib fractures after being stomped by a horse. Management included intercostal drainage placement, costal fixation from the 5th to the 10th rib, and intensive care unit admission. Postoperative complications included, acute kidney injury, rhabdomyolysis, and severe acute respiratory distress syndrome. This case highlights the importance of a multidisciplinary approach in managing complex traumatic injuries and emphasizes potential complications despite optimal care.

## Introduction

Traumatic hemothorax is a serious medical condition that arises when blood accumulates in the pleural cavity due to chest trauma, most commonly from blunt force injuries such as motor vehicle accidents, falls, or assaults [1]. This condition carries significant morbidity and mortality, primarily because it compromises respiratory function and can lead to hypovolemic shock if not promptly addressed [2]. Early detection and intervention are essential to prevent complications such as infection, retained hemothorax, and progressive respiratory failure [3].

This case report details a traumatic hemothorax secondary to a horse stomping incident, emphasizing the unique challenges in both diagnosis and management. Animal-related injuries, particularly those caused by large animals like horses, are uncommon but can cause severe chest trauma due to the sheer force involved, often leading to complex injury patterns, including rib fractures, pulmonary contusions, and hemothorax [4,5]. These injuries are further complicated by potential multisystem involvement, such as abdominal or orthopedic trauma, demanding a multidisciplinary approach [6].

## Case presentation

A 48-year-old professional horse rider presented to the emergency department on December 29, 2023, after being trampled on the chest by a horse the previous day. The patient reported immediate chest pain and difficulty breathing following the incident. Upon arrival, initial vital signs included a blood pressure of 101/62 mmHg, heart rate of 78 beats per minute, respiratory rate of 19 breaths per minute, temperature of 36.9°C, and oxygen saturation of 94%.

The patient had no significant medical history, no prior surgeries, and no known allergies. His blood type was unknown. He had a long-standing history of smoking, consuming between five and 15 cigarettes per day since the age of 17, and drinking alcohol occasionally, approximately 500 ml of beer.

**Figure 1:**
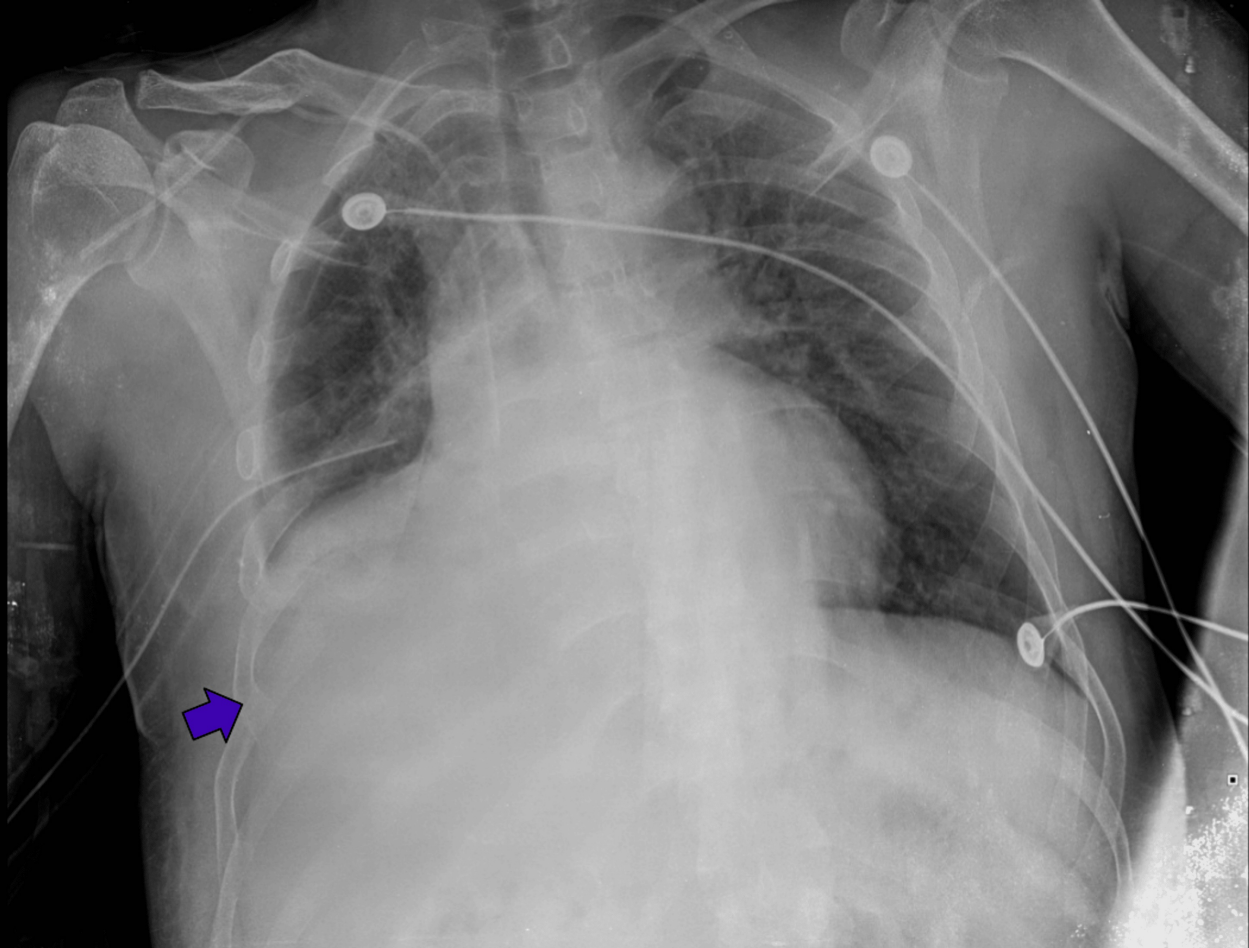
First posteroanterior (PA) chest X-ray on admission. Despite the rotation of the X-ray, right pleural effusion with poor aeration and increased lung density (blue arrow) is appreciated, likely due to contusion or edema. A jugular catheter with the tip in the right atrium and intercostal tube at the lung base and the right rib fractures from the fifth to the 12th rib were noted.

The patient arrived at the hospital after an initial evaluation at an external emergency unit. An intercostal drainage (ICD) was immediately placed, initially draining 350 cc of blood. Upon arrival at our center, total drainage over the next 24 hours reached 1000 cc. However, no follow-up X-ray was initially performed, necessitating one (Figure 1), which revealed right pleural effusion with poor aeration and increased lung density of the right hemithorax, consistent with a significant hemothorax. Due to its unavailability in the initial emergency unit, subsequent imaging and laboratory tests showed anemia, elevated creatine phosphokinase (CPK), and hypercreatininemia (Table 1).

**Table 1:** Laboratory results. Laboratory tests showed low hemoglobin, indicating anemia due to blood loss. Elevated CPK suggests muscle damage and increased creatinine points to possible acute kidney injury. These findings highlight significant physiological stress.

Parameter	Result	Unit	Reference range
Hemoglobin	11.2	g/dL	13.8-17.2
Creatine phosphokinase (CPK)	1600	U/L	55-170
Creatinine	1.6	mg/dL	0.7-1.3

Surgical procedure and rib fixation

Given the severity of the rib fractures and the instability of the chest wall, a surgical intervention was deemed necessary.

**Figure 2:**
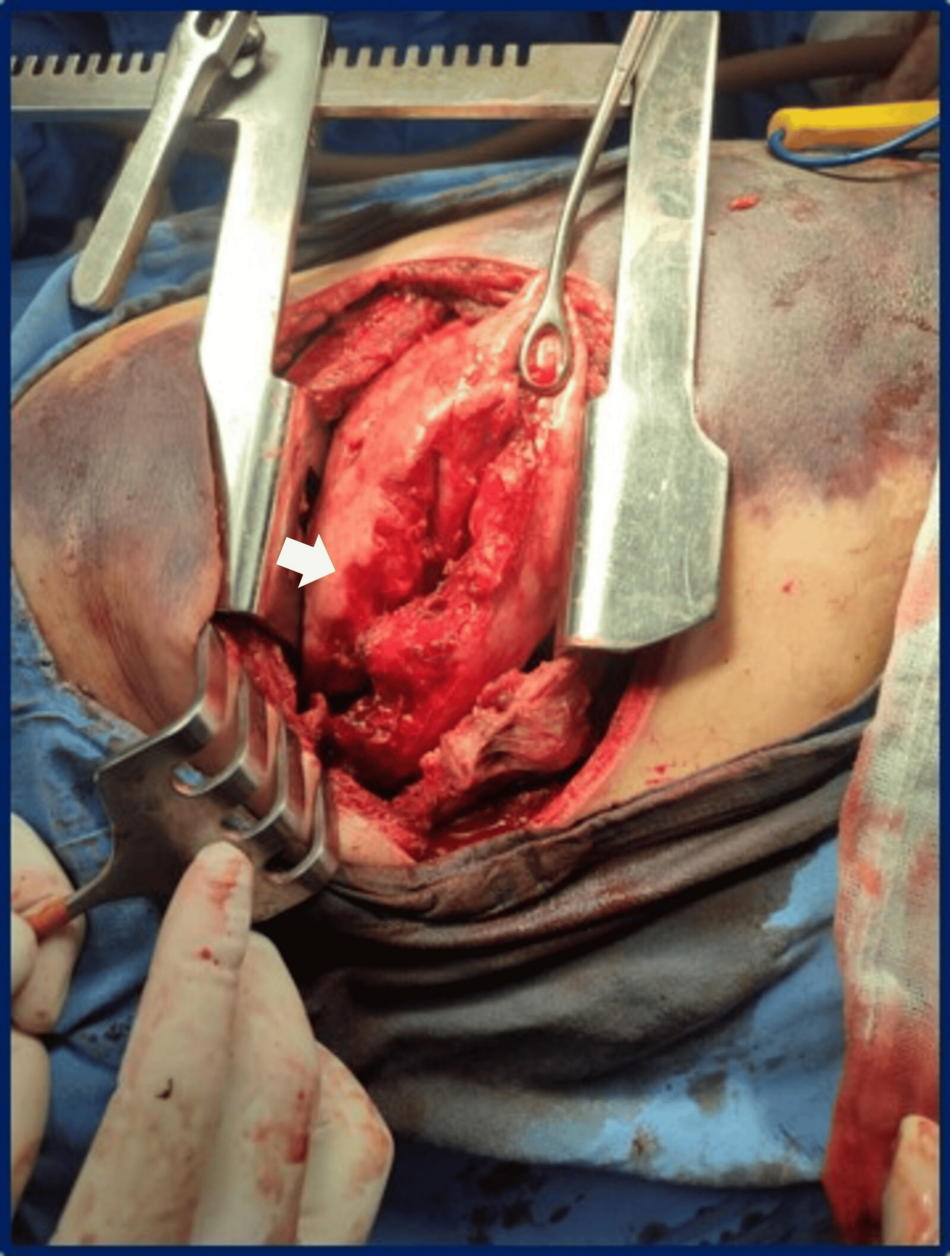
Laceration in the lower lobe. Grade IV laceration of the lower lobe (white arrow), according to the American Association for the Surgery of Trauma (AAST) pulmonary injury scale [7].

Following a safe surgery protocol and under general anesthesia, the patient was positioned in the left lateral decubitus position, with ventilation managed via a tracheostomy due to upper airway inflammation. After asepsis, antisepsis, and preparation of the sterile field, a right posterolateral thoracotomy was performed, accessing the thoracic cavity through the 6th intercostal space. Upon entering the pleural cavity, approximately 300 cc of retained hemothorax was evacuated. Basal pleuropulmonary adhesions were lysed, and parenchymal release revealed a 10-cm grade IV laceration in the lower lobe (Figure 2).

**Figure 3:**
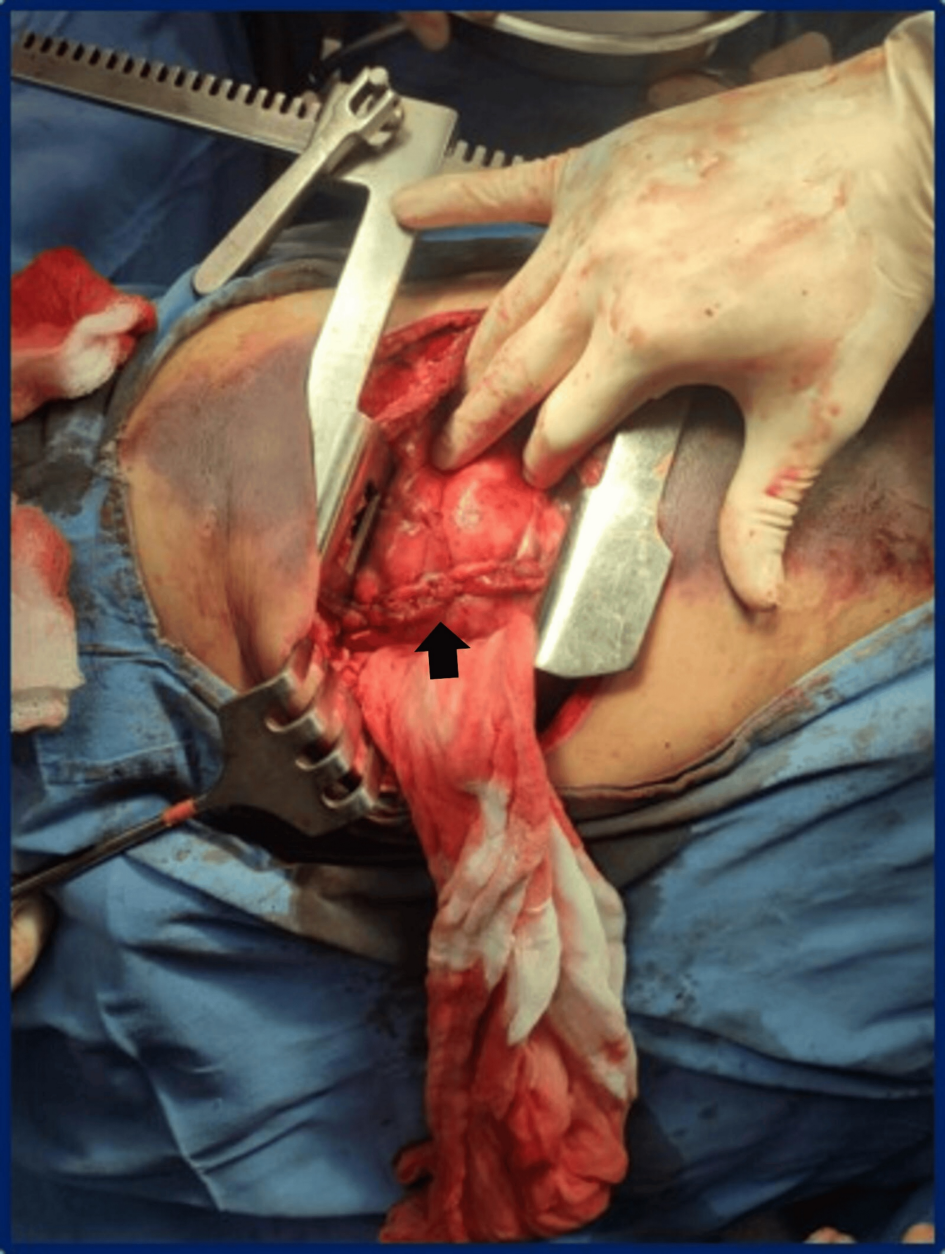
Closure with tractotomy and double-layer sutures. Tractotomy of the lesion, followed by closure with double-layer sutures (black arrow). Pulmonary tractotomy is a surgical procedure primarily used in the management of traumatic lung injuries. In this technique, a linear stapler is inserted through the wound in the pulmonary parenchyma, dividing the lung tissue between the bleeding points. This approach allows direct access to the bleeding vessels, facilitating their control while minimizing the loss of healthy lung tissue.

A tractotomy was performed to manage the injury, followed by closure with double-layer sutures using 2-0 polyglycolic acid (Figure 3).

**Figure 4:**
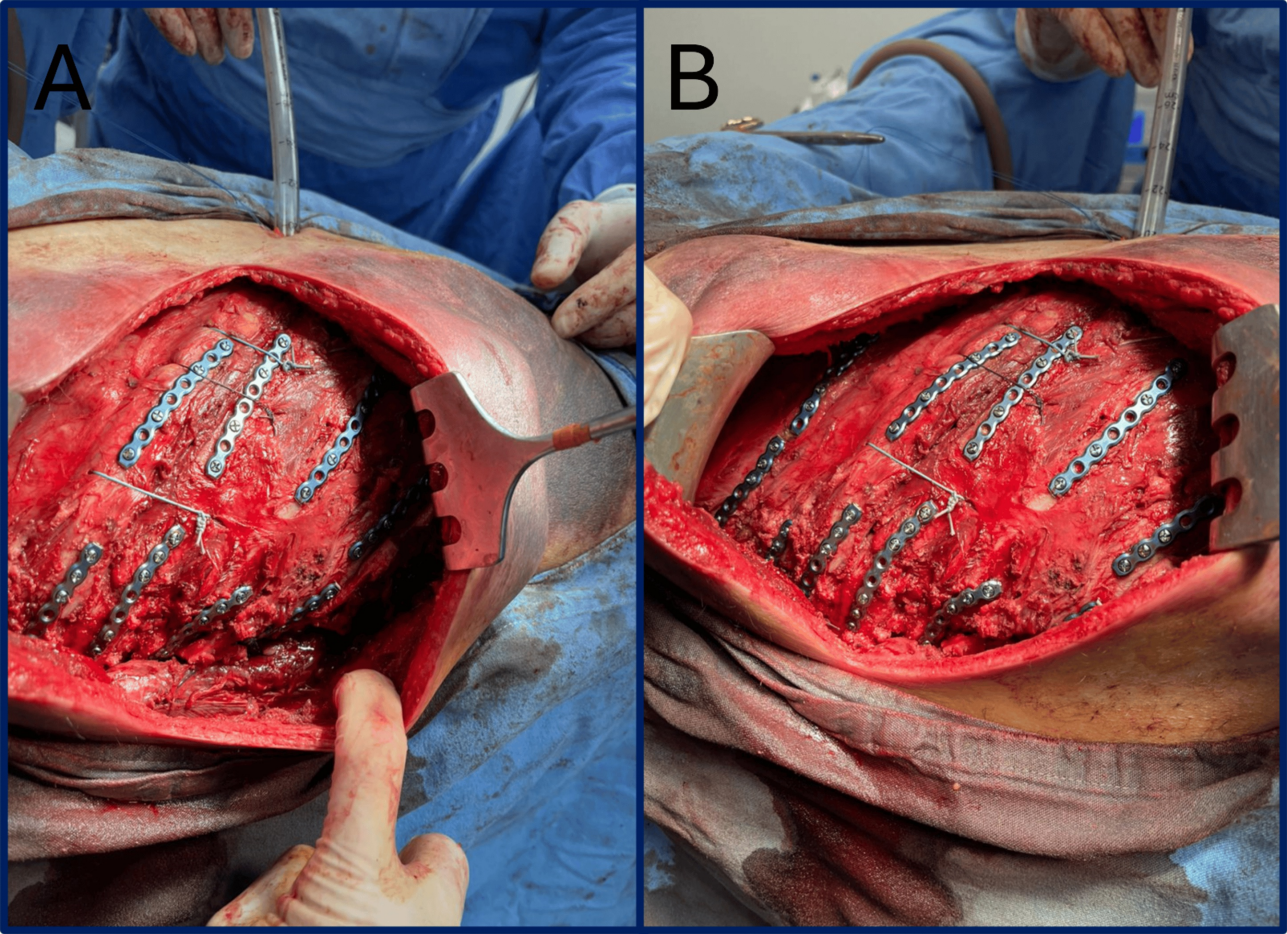
Open reduction and internal fixation. Open reduction and internal fixation of the 5th to 10th ribs (A and B) with titanium plates and locking screws.

Hemostasis and the absence of air leaks were confirmed. The rib fractures were surgically exposed by elevating the periosteum adjacent to each fracture site. Open reduction and internal fixation were performed on ribs five to 10 using titanium plates and locking screws. Intercostal stability and hemostasis were verified (Figure 4). A 36 Fr intercostal drain was placed through a contralateral incision in the eighth intercostal space and secured. The ribs were approximated using 1-0 polyglycolic acid sutures, and the muscle layers were closed with 2-0 polyglycolic acid sutures. Skin closure was achieved with staples.

During the procedure, the patient received a red blood cell transfusion, an epidural block, and an epidural infusion pump placed by the anesthesiology team. The patient was transferred to the ICU postoperatively.

Postoperative care and complications

Postoperative management included advanced airway support with mechanical ventilation in volume-controlled mode to maintain adequate oxygenation and ventilation. The patient’s fluid and electrolyte balance was closely monitored and managed to prevent complications such as acute kidney injury and rhabdomyolysis. Pharmacological interventions included the use of analgesics, antibiotics, and gastrointestinal protection with omeprazole.

The endopleural tube was removed three days later due to a total output of 90 cc of serohematic content.

Despite aggressive surgical and postoperative management, the patient developed acute respiratory distress syndrome (ARDS) and required continuous hemodynamic support with vasopressors. The patient’s condition remained critical with a poor prognosis due to the severity of the initial injuries and subsequent complications, such as surgical site infection or persistent hemopneumothorax.

Radiographic follow-up (two months postoperatively)

**Figure 5:**
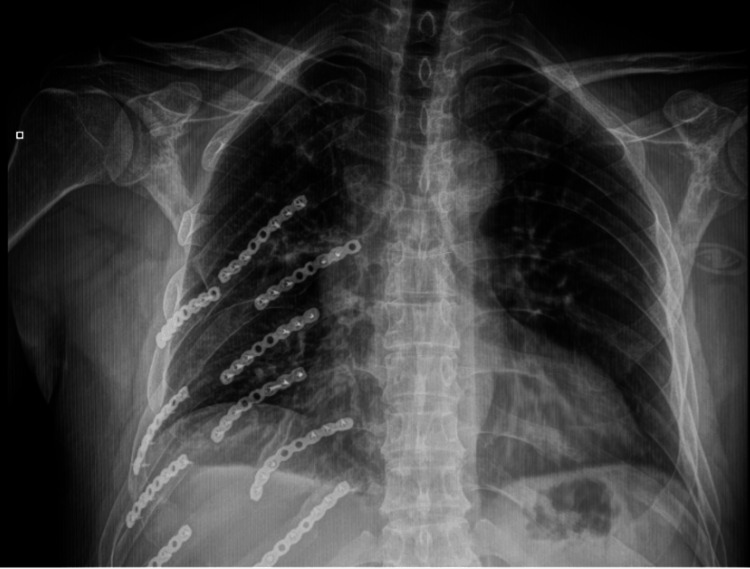
Follow-up posteroanterior chest radiograph taken two months after surgery. Posteroanterior chest X-ray showing improved bilateral lung aeration. Persistent perihilar infiltrates and basal atelectasis in the right lung are noted. Osteosynthesis material in the right rib cage is in the proper position.

A follow-up chest X-ray taken two months after surgery showed multiple titanium plates and screws along the right rib cage, corresponding to the previously described rib fixation from the 5th to the 10th rib (Figure 5). These fixation devices were correctly aligned and showed no evidence of displacement or loosening, indicating stable healing of the rib fractures.

There was no visible recurrence of the hemothorax, and the pleural spaces appear clear, with no signs of fluid accumulation or pneumothorax. Both lungs were well-expanded, with normal parenchymal markings, suggesting adequate respiratory recovery. The cardiovascular and mediastinal structures were in their expected anatomical positions, with no evidence of displacement or mediastinal compression. The alignment and integrity of the chest wall were well-preserved, confirming the successful stabilization achieved through surgical intervention.

This radiographic follow-up confirmed favorable postoperative healing, with satisfactory stabilization of rib fractures and no radiological signs of complications such as infection, hardware failure, or recurrent pleural pathology.

## Discussion

The management of a traumatic hemothorax complicated by multiple rib fractures, as described in this case, exemplifies the challenges posed by severe thoracic trauma. Traumatic hemothorax, often resulting from blunt trauma, requires rapid intervention to evacuate blood from the pleural space and prevent respiratory and hemodynamic compromise. Placement of a thoracic tube (tube thoracostomy) is the first line of treatment to drain the accumulated fluid, reduce pleural pressure, and facilitate lung expansion. Failure to promptly address these issues can result in retained hemothorax or infection [1,2,5].

Rib fixation has gained prominence in managing multiple rib fractures with significant chest wall instability. Fixation with titanium plates and screws helps restore structural stability, reduces the need for mechanical ventilation, and lowers the incidence of post-trauma complications like pneumonia [4,8]. Surgical fixation also leads to faster recovery and reduced intensive care unit stay, which aligns with modern recommendations for managing flail chest and multiple rib fractures [3,9,10].

Despite optimal surgical and postoperative care, patients with severe chest trauma are at high risk for complications like ARDS. ARDS is associated with widespread pulmonary inflammation and requires protective mechanical ventilation strategies to optimize oxygenation while minimizing ventilator-induced lung injury [6,11]. In the presented case, ARDS development reflects the severity of trauma and the systemic inflammatory response induced by injury.

In addition to respiratory challenges, the patient developed acute kidney injury secondary to rhabdomyolysis, a condition resulting from muscle breakdown and myoglobin release, which can overwhelm renal clearance. Careful management of fluids and electrolytes is crucial in preventing further renal deterioration in such cases [1,2]. The ICU environment provided the necessary monitoring and intervention to manage these complications effectively.

The two-month follow-up confirmed satisfactory recovery, with proper alignment of rib implants and no evidence of complications like infection or hardware failure. This favorable outcome highlights the value of rib fixation in improving the mechanical function of the thoracic cage and facilitating faster recovery after trauma.

## Conclusions

Severe traumatic hemothorax from blunt chest trauma demands prompt and aggressive management to prevent life-threatening complications. Without timely intervention, significant blood loss can lead to hypovolemic shock, while lung injury or hypoxia may result in ARDS. Cardiac tamponade and tension pneumothorax, caused by blood or air accumulation, can further compromise cardiac and respiratory function. Persistent bleeding can worsen coagulopathy, while infectious complications, such as empyema or pleural sepsis, pose additional risks. Pulmonary complications, including atelectasis and fibrothorax, may impair long-term respiratory function. Early diagnosis, proper drainage, and hemodynamic stabilization are crucial to preventing these complications and improving patient outcomes.
